# Single-Trial Classification of Error-Related Potentials in People with Motor Disabilities: A Study in Cerebral Palsy, Stroke, and Amputees

**DOI:** 10.3390/s22041676

**Published:** 2022-02-21

**Authors:** Nayab Usama, Imran Khan Niazi, Kim Dremstrup, Mads Jochumsen

**Affiliations:** 1 Department of Health Science and Technology, Aalborg University, 9000 Aalborg, Denmark; nu@hst.aau.dk (N.U.); imrankn@hst.aau.dk (I.K.N.); kdn@hst.aau.dk (K.D.); 2 Centre for Chiropractic Research, New Zealand College of Chiropractic, Auckland 1060, New Zealand; 3 Health and Rehabilitation Research Institute, AUT University, Auckland 0627, New Zealand

**Keywords:** error-related potentials, brain-computer interface, cerebral palsy, amputation, stroke, neurorehabilitation, artificial neural network

## Abstract

Brain-computer interface performance may be reduced over time, but adapting the classifier could reduce this problem. Error-related potentials (ErrPs) could label data for continuous adaptation. However, this has scarcely been investigated in populations with severe motor impairments. The aim of this study was to detect ErrPs from single-trial EEG in offline analysis in participants with cerebral palsy, an amputation, or stroke, and determine how much discriminative information different brain regions hold. Ten participants with cerebral palsy, eight with an amputation, and 25 with a stroke attempted to perform 300–400 wrist and ankle movements while a sham BCI provided feedback on their performance for eliciting ErrPs. Pre-processed EEG epochs were inputted in a multi-layer perceptron artificial neural network. Each brain region was used as input individually (Frontal, Central, Temporal Right, Temporal Left, Parietal, and Occipital), the combination of the Central region with each of the adjacent regions, and all regions combined. The Frontal and Central regions were most important, and adding additional regions only improved performance slightly. The average classification accuracies were 84 ± 4%, 87± 4%, and 85 ± 3% for cerebral palsy, amputation, and stroke participants. In conclusion, ErrPs can be detected in participants with motor impairments; this may have implications for developing adaptive BCIs or automatic error correction.

## 1. Introduction

Brain-computer interfaces (BCIs) provide individuals with severe motor impairments the possibility to control external devices using only brain activity [1,2,3]. Examples of such devices could be wheelchairs and robotic manipulators for mobility restoration, speller devices for communication, and electrical stimulators or rehabilitation robots for motor rehabilitation after e.g., stroke [1,4,5]. Various control signals can be used to control BCIs, such as steady-state visually evoked potentials [2,6], P300, movement-related cortical potentials, and sensorimotor rhythms. These control signals are recorded from the electrical activity of the brain and processed to enhance the signal-to-noise ratio, after which they are detected/classified and translated into device commands. To ensure good performance of the BCI, several factors need to be attended to such as proper electrode montage and impedances for recording the brain activity and good calibration data for the classifier [7,8]. The calibration data that often are recorded prior to the actual use of the BCI may not represent the actual brain activity well after the BCI has been used for some time, e.g., due to changes in electrode impedance or if the user starts to fatigue. This problem could be accounted for if the classifier in the BCI is continuously updated. Error-related potentials (ErrPs) have been proposed as a means for this [9]. An ErrP is elicited when a person realizes an error e.g., the output of the BCI is different than expected. With proper ErrP detection, erroneously classified data can be correctly labelled and the classifier in the BCI can be correctly updated based on the most recent data. Another application of ErrPs within BCI is error correction, where an erroneous action of the BCI, e.g., in a P300 speller or movement of a robotic arm, can be detected and the incorrect action can be reverted automatically [9,10]. If the ErrPs are properly detected, the performance of the BCI can be improved, since the potential errors do not need to be corrected manually (see e.g., [11,12,13]). It has been shown in several studies that ErrPs can be detected from single-trial EEG [9,10], primarily from able-bodied individuals, but only a few studies have investigated the detection of ErrPs in individuals with movement disabilities. It has previously been shown how various factors modulate the ErrP in stroke patients [14,15], and that ErrPs in stroke patients can be detected from single-trial EEG [16]. In addition, it has been reported that ErrPs can be elicited and detected in individuals with spinal cord injury [13,17,18], amyotrophic lateral sclerosis [19], and epilepsy [20,21]. Lastly, error processing has been investigated in individuals with Parkinson’s disease [22] and cerebral palsy [23], but detection of ErrPs in these conditions has not been performed. ErrPs have generally been detected using temporal waveform features of a bandpass filtered epoch (~0–1 s after the feedback of the outcome) from electrodes on the scalp in the proximity of the anterior cingulate cortex amongst other neural generators (roughly around FCz according to the 10–20 EEG system) [9,10]. ErrPs can be detected from a single or few electrodes around FCz [24,25], but studies have reported that additional discriminative information can be obtained from using more electrodes covering other parts of the brain [24,26,27,28,29,30,31,32,33,34,35]. The aim of this study was twofold; first it was investigated whether ErrPs could be detected in individuals with motor disabilities after cerebral palsy, an amputation, or stroke in offline analysis, and secondly, how much discriminative information different brain regions bring to the detection of ErrPs.

## 2. Materials and Methods

### 2.1. Participants

In this study, ten participants with cerebral palsy (for clinical characteristics see Table 1), eight amputees (see Table 2), and 25 participants with a stroke (see Table 3) were recruited. The experiments were conducted at Allied Hospital, Faisalabad, Pakistan. All participants or their parents provided written informed consent before the experiment. The procedures were approved by the local ethical committee at Allied Hospital and were conducted according to the Helsinki Declaration. The cerebral palsy participants were recruited through the Department of Pediatrics, amputees were enlisted through the Department of Orthopedics, and stroke participants were recruited at the Department of Neurology at Allied Hospital Faisalabad. All the cerebral palsy participants were diagnosed between ages of 1–3 years. The cerebral palsy participants’ motor abilities were assessed by a pediatrician at Allied Hospital in terms of the gross motor function classification system (GMFCS), (I = ambulatory, II = some limitations in motor functions, III = dependent on others or some assistive devices). The motor abilities of the stroke participants were assessed by a Neurologist in terms of the Bruunstrom Stage classification. The data from the stroke patients have been presented in a recent study, but the experimental details are described in detail in the following sections [16].

### 2.2. Data Recording

EEG was recorded from 64 channels with active electrodes with a sampling rate of 1200 Hz (g.HIamp and g.GAMMASYS, G.Tec, Graz, Austria). The electrodes were placed according to the 10-10 system and were grounded to AFz and referenced to a linked ear reference. During the experiment the electrode impedances were below 10 kΩ. The EEG was synchronized to visual cues through an Arduino controller from a custom-made MATLAB script (MathWorks^®^, Natick, MA, USA) which sent a trigger to the EEG amplifier. The external triggers were used to divide the continuous EEG into epochs containing error and correct responses.

### 2.3. Experimental Details

The participants were seated in a comfortable chair in front of a computer screen which displayed the visual cues throughout the experiment. The experiment consisted of 15 runs for the participants with cerebral palsy and 20 runs for the amputees and stroke participants, where each run consisted of 20 trials. Each trial started with an idle phase lasting five seconds where the participant could relax, this was followed by a preparation phase lasting three seconds where the participant was cued to bring the attention back to the screen and prepare to attempt to perform a movement. In the movement phase a picture of the hand or foot was shown pointing to the left or right indicating that a wrist extension of the right or left hand should be performed or a dorsiflexion of the right or left foot. The movement phase lasted three seconds for the amputees and stroke participants and five seconds for the participants with cerebral palsy to allow them more time to process what intended movement that should be attempted. The amputees were instructed to imagine the movement of their amputated limb. A single movement attempt was performed in each movement phase. After the movement phase, visual feedback with a ratio of 70/30 (for correct/ incorrect) was presented as a green coloured tick mark or a red coloured cross sign indicating whether the movement was correctly or incorrectly detected based on the brain activity. No actual detection of movements was performed, but it was conveyed to the participant that a BCI classified the movements [16,36]. During the feedback monitoring the participant was instructed to avoid unnecessary movements and eye blinks. An equal number of 75, 100, and 100 movements were performed for the left/right hand and foot for the cerebral palsy, amputees, and stroke participants respectively, i.e., 300, 400, and 400 movements in total. At the end of each run a break was given until the participant was comfortable with resuming the experiment. The experiments were completed in approximately 100–180 min.

### 2.4. Signal Processing

#### 2.4.1. Pre-Processing

The continuous EEG data were bandpass filtered between 1–10 Hz with an 8th order zero phase-shift Butterworth filter. After the filtering, bad channels and epochs were rejected from the analysis. Channels having a mean amplitude more than three standard deviations above the overall mean amplitude across all channels were defined as bad channels and removed. Next, the filtered data were divided into 0.7 s epochs (starting from 0.1 s after the presentation of the visual feedback, i.e., green tick mark or red plus sign, until 0.8 s after) to capture the negative and positive peaks of the error and correct responses. Bad epochs were defined as an epoch with peak-peak amplitude exceeding ±150 µV. To have an equal number of error and correct responses, random correct responses were selected for the further data analysis to match the number of error responses. The data analysis was performed in MATLAB (MathWorks^®^).

#### 2.4.2. Classification

For the classification of error and correct responses a multi-layer perceptron artificial neural network (MLP ANN) was used. In a recent study [16], we found that MLP ANN performed better than a linear discriminant analysis classifier, which is often used for classifying ErrPs. Therefore, we chose to perform the classification with MLP ANN.

**Figure 1:**
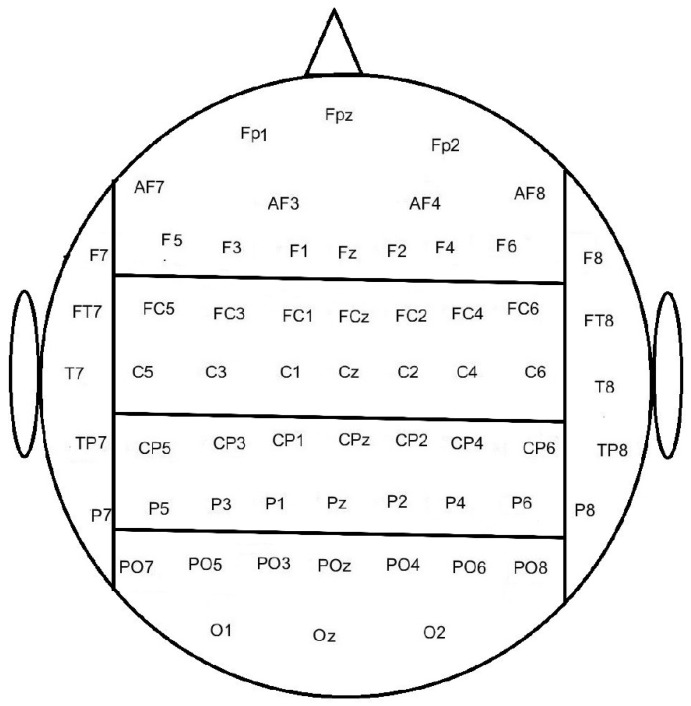
The electrodes were divided into Frontal, Central, Parietal, Occipital, Temporal Left, and Temporal Right brain regions. Note that there is a different number of electrodes in the different brain regions.

The input for the MLP ANN was the entire pre-processed epoch. The MLP ANN had 5 layers where the input, layer 1, was the data points in the epoch for the channels of interest (dimension: number of channels x number of samples in epoch). Three hidden layers were used, they had a size of 100-50-25, whereas the output layer was of size 1 with a sigmoid activation function. The MLP ANN was trained using the scaled conjugate gradient descent method, and the performance of the MLP ANN was validation checked with cross-entropy. The classification was performed with different electrode configurations to investigate how well error responses can be discriminated from correct responses. The electrodes were divided into six specific brain regions: Frontal, Central, Parietal, Occipital, Temporal Left, and Temporal Right (see Figure 1). Initially, each region was used as input for the MLP ANN individually. We had a hypothesis about ErrP classification being highest at the Central brain region based on existing ErrP literature and the proximity of the anterior cingulate cortex. Thus, the classification was performed again with the Central region as input combined with each of the adjacent brain areas (i.e., Frontal, Parietal, Temporal Left, and Temporal Right). Next, the classification was performed with all brain regions except the Occipital region, and lastly, the classification was performed with all brain regions combined. In all the classification scenarios, 10-fold cross-validation was used, the same folds were used across the different classification scenarios. The analyses were performed using MATLAB (MathWorks^®^).

## 3. Results

On average, 0.6 ± 0.7 channels (range: 0–2) and 65 ± 56 epochs (range: 9–141) were excluded for the cerebral palsy participants, 0.5 ± 1.4 channels (range: 0–4) and 59 ± 71 epochs (range: 0–191) were excluded for the amputees, and 0.3 ± 0.5 channels (range: 0–1) and 72 ± 64 epochs (range: 2–228) were excluded for the stroke participants.

**Figure 2:**
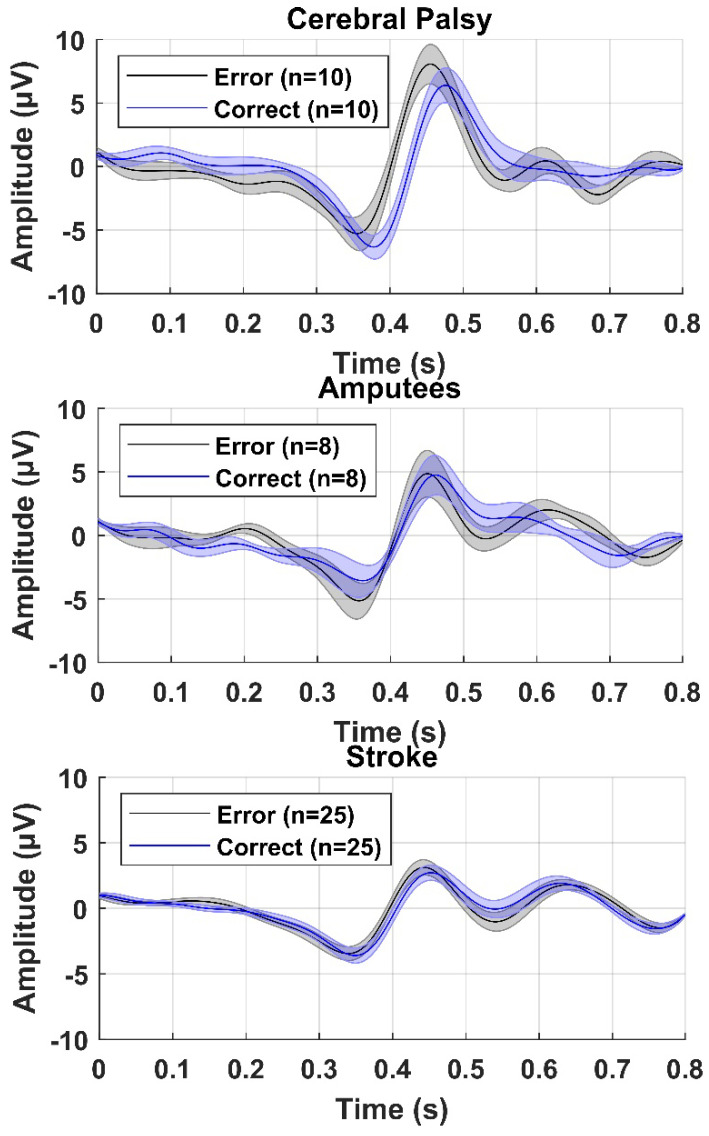
Grand average of error and correct responses from FCz across the ten participants with cerebral palsy (**top**), eight participants with an amputation (**middle**), and 25 participants with a stroke (**bottom**). The solid line is the mean across participants and the shaded area represents the standard error.

**Figure 3:**
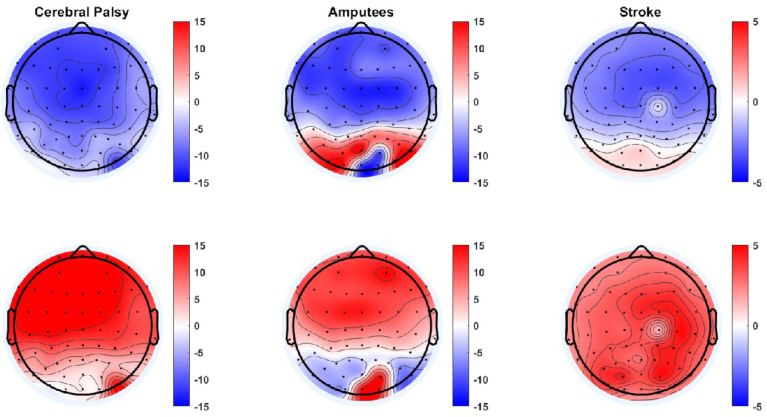
Topographical plot of the negative (**top**) and positive (**bottom**) peaks of the error responses for a representative participant with cerebral palsy (**left column**), a participant with an amputation (**middle column**), and a participant with a stroke (**right column**). The unit of the color bar is in µV.

The average error and correct responses are presented in Figure 2 for participants with cerebral palsy, participants with an amputation, and participants with a stroke. From the averages it can be seen that there was a consistent negative peak between 0.3 and 0.4 s after the presentation of the feedback and a positive peak 0.1 s after the negative peak. Based on the grand averages, there was a slightly higher peak-peak amplitude between the negative and positive peaks for the error responses compared to the correct responses. In Figure 3, topographical plots are shown from representative participants. It can be seen that the Central and Frontal channels show the most negative and positive peaks, although most other channels also show a similar negative or positive peak, but with smaller amplitudes. In the following sections, the classification accuracies are presented as mean ± standard error across participants.

**Figure 4:**
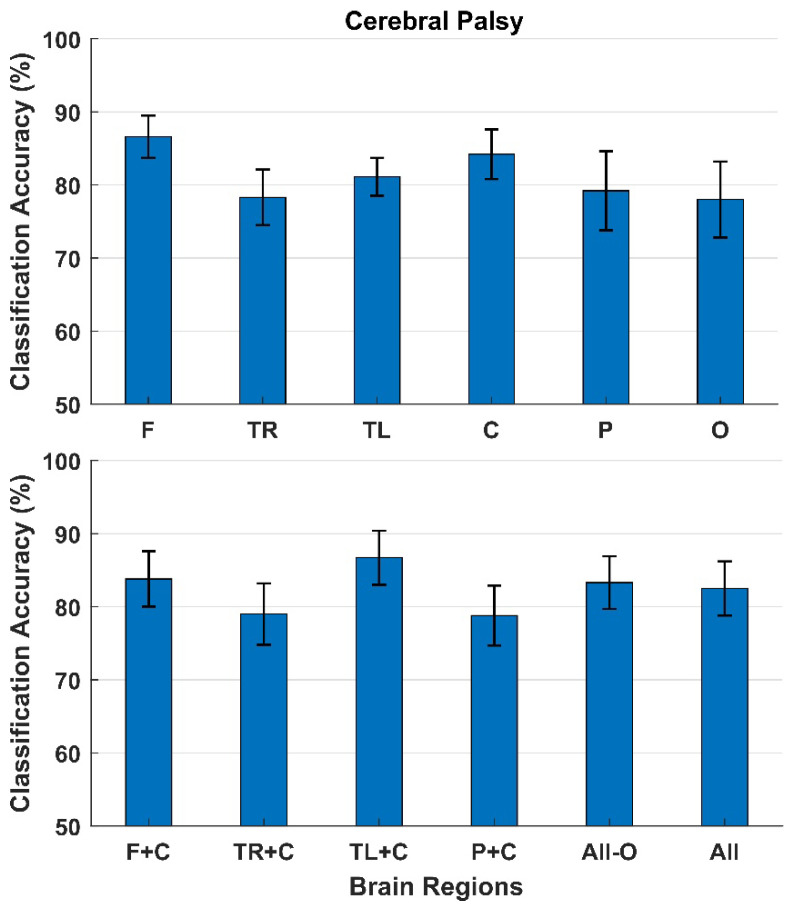
Classification between error and correct responses. The bars represent the mean across participants with cerebral palsy, and the standard error is shown as well. The top of the figure shows the classification accuracies obtained using each brain region individually, and the bottom of the figure shows the classification accuracies when combining different brain regions. F: Frontal, TR: Temporal Right, TL: Temporal Left, C: Central, P: Parietal, and O: Occipital.

The classification results for participants with cerebral palsy are presented in Figure 4. With a single brain region as input, the highest classification accuracies between error and correct responses were obtained with the electrodes from the Frontal region (87 ± 3%) followed by the Central region (84 ± 3%). The lowest classification accuracies were obtained from the Occipital region (78 ± 5%). The classification accuracies did not increase when combining different regions.

**Figure 5:**
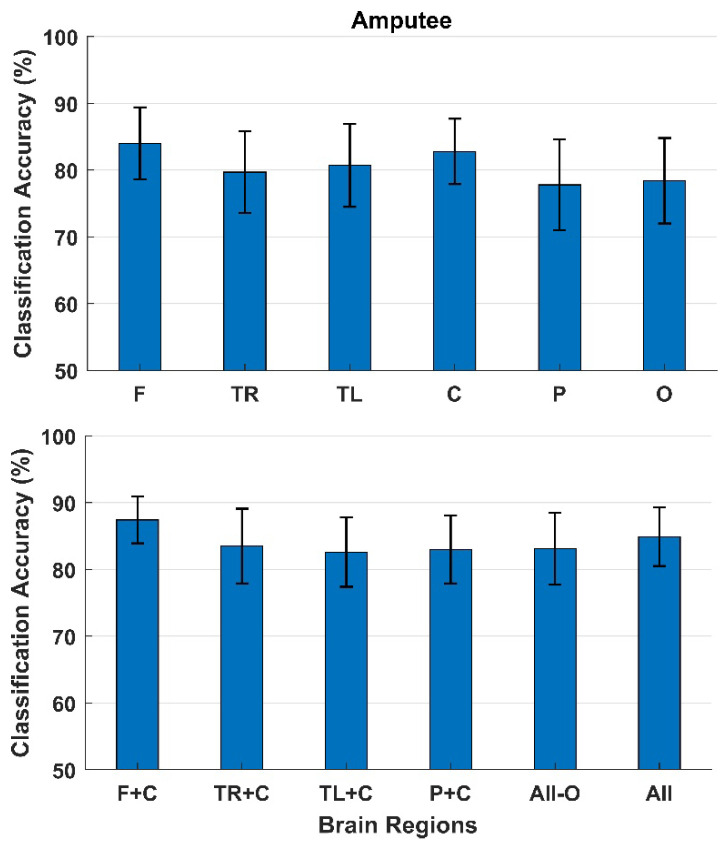
Classification between error and correct responses. The bars represent the mean across participants with an amputated limb, and the standard error is shown as well. The top of the figure shows the classification accuracies obtained using each brain region individually, and the bottom of the figure shows the classification accuracies when combining different brain regions. F: Frontal, TR: Temporal Right, TL: Temporal Left, C: Central, P: Parietal, and O: Occipital.

The classification results for participants with an amputation are presented in Figure 5. As with the cerebral palsy patients, the highest classification accuracies with a single brain region as input were obtained with the electrodes from the Frontal region (84 ± 5%) followed by the Central region (83 ± 5%). The lowest classification accuracies were obtained from the Parietal region (78 ± 7%). The classification accuracies increased when adding the Frontal region to the Central region (87 ± 4%); however, when adding more regions, the classification accuracies decreased (85 ± 4%).

**Figure 6:**
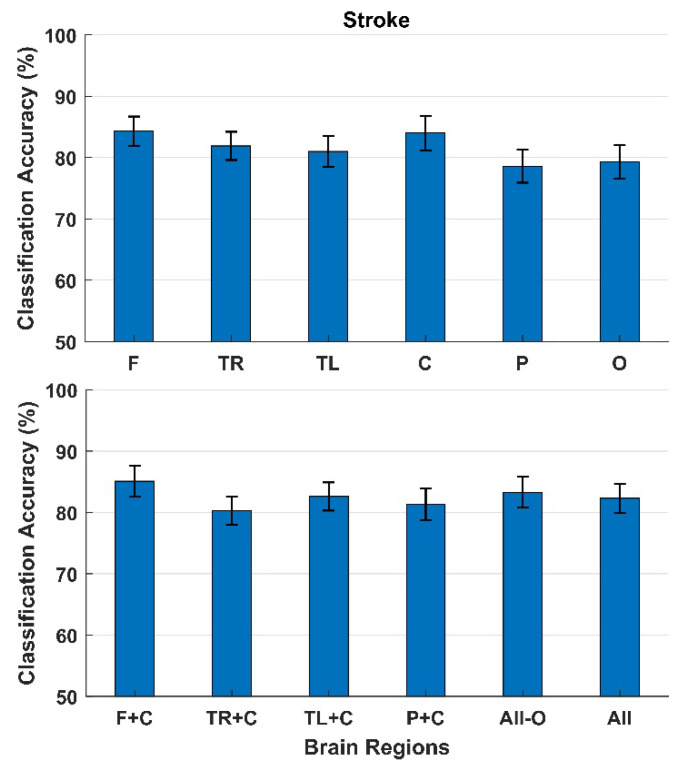
Classification between error and correct responses. The bars represent the mean across participants with a stroke, and the standard error is shown as well. The top of the figure shows the classification accuracies obtained using each brain region individually, and the bottom of the figure shows the classification accuracies when combining different brain regions. F: Frontal, TR: Temporal Right, TL: Temporal Left, C: Central, P: Parietal, and O: Occipital.

The classification results for participants with a stroke are presented in Figure 6. Like the cerebral palsy and amputation participants, the highest classification accuracies with a single brain region as input were obtained with the electrodes from the Frontal (84 ± 2%) and Central region (84 ± 3%). The lowest classification accuracies were obtained from the Parietal region (79 ± 3%). The classification accuracies increased slightly when combining the Frontal and Central region (85 ± 3%).

## 4. Discussion

In this study it was shown that error responses can be discriminated from correct responses from single-trial EEG recordings in participants with cerebral palsy, an amputation, and a stroke. The highest classification accuracies were obtained from the Frontal and Central brain regions from all participant groups with average classification accuracies of 84 ± 4%, 87 ± 4%, and 85 ± 3% for cerebral palsy, amputees, and stroke participants, respectively. This was also expected, since the Frontal and Central electrodes from the scalp are closest to the main neural generators associated with error processing such as the anterior cingulate cortex [37]. The classification accuracies only increased slightly when information from other brain areas were added to the Frontal and Central region. However, the classification accuracies from the different regions individually were all significantly higher than chance level, calculated with a significance level of 5% (between 62 and 66% for amputees/stroke and cerebral palsy, respectively) [38]. This could indicate that there is a high correlation between electrodes, which could be due to the fact that the EEG correlates of the error/correct responses are widely distributed over the scalp, potentially due to volume conduction.

The findings agree with several other studies that have reported the midline electrodes (especially FCz and Cz) to contain the highest error-related activity [24,28,29,30,31,34,35,39,40], but also Parietal areas [24,26,27,28,31,32,33,35] and the Occipital cortex have been associated with error-related activity [27,28,33,35]. It has been reported that the ErrP has the highest amplitudes around the midline channels but is still visible in the channels in the periphery furthest away from the midline, with a smaller amplitude though [27,33,34,35]. This may explain why all brain regions individually provide enough discriminative information to provide classification accuracies that are significantly higher than chance level; however, it has also been reported that the channels in the periphery lead to classification accuracies around chance level [29]. The fact that the ErrP can be observed in all channels, but with smaller amplitudes in the periphery, may also explain why adding additional regions to the Frontal and Central regions does not provide much additional discriminative information to the classification. This finding is also supported by previous studies that have found similar marginal increments/decrements in classification accuracies when multiple brain regions are used as input for classification [28,30]. It should also be noted that there is a different number of electrodes in the different brain regions in this study, which in itself could affect the classification accuracies although their spatial distribution probably matters most. It has been reported that the classification accuracies increase with a higher number of electrodes used for classification of ErrPs and motor imagery [29,33,41].

Despite this focus on populations with motor impairments, cerebral palsy, amputation, and stroke, it was still possible to decode ErrPs with accuracies similar to what has been reported previously in studies with able-bodied participants and participants with other types of motor impairments. This could be expected since the ErrP is elicited by the perception of an error, although the motor impairment could potentially cause lower expectations of one’s own performance and hence affect the elicitation of the ErrP if the participant does not expect to be able to succeed in the task. However, this was not probed in the current study. The classification accuracies in similar studies have been reported to be in the range of 70–90% see e.g., [16,24,30,36,39,42,43,44,45,46,47,48]. However, it should be noted that bad epochs were rejected as part of the pre-processing in the current study, which is likely to have improved the decoding. These results may be optimistic as to what can be obtained in online decoding of errors. The approach of using MLP ANN has previously been shown to be useful for decoding ErrPs in stroke patients [16], and with this approach it is not necessary to extract features, since the entire epoch is used as input for the classifier. However, this classifier showed poor between-day and across-participant transfer [16], which suggests that calibration data for the classifier need to be collected every time it is going to be used. This can be a time-consuming process and it should be considered whether other, more generic approaches should be used [43,49,50,51], that can be individualized/adapted [17,52] to the user while an error monitoring/correction system is in use. Alternatively, it should be considered or whether a type of ErrP should be used in which multiple trials can be obtained more rapidly, such as observational ErrPs [53], or with higher error/correct ratios [25,54]. These considerations should be tested in future studies where online control of a BCI with error correction should be evaluated e.g., for error correction in myoelectric prosthetic control for people with an amputation, control of assistive devices such as wheelchairs, speller devices, or games for people with cerebral palsy or for adaptation of BCIs for stroke rehabilitation to account for fatigue or shifts in attention.

In future studies it could be relevant to perform source localization to better understand the origin of the error-related activity and investigate how it differs from the processing of feedback in general: i.e., the correct responses. It would also be relevant to perform channel selection to identify the optimal channels for identifying error-related activity and to investigate how much the relevant channels differ between users and across conditions. Another aspect that could be investigated is how to further optimize the decoding of ErrPs, which could be done using various techniques such as signal decomposition techniques and blind source separation for pre-processing the signals [55,56]. In this study, we only employed one specific type of neural network, but it is likely that other neural networks or tuning of them could yield better performance [57,58]. This could be tested systematically with different neural networks with inherent feature extraction, so no feature extraction with a priori knowledge is needed. Ideally, the classifier should have good generalization properties across days and users to avoid extensive calibration of it with user-specific data.

The results in this study also indicated that ErrPs could be decoded from the periphery, e.g., from non-hairy electrode locations around the ear. It could potentially allow the use of a more aesthetically appealing headset/electrode setup that also would not require hair wash after each use, which would be an important consideration for permanent BCI users [59,60]. This could be investigated in future studies.

## 5. Conclusions

In this study it was shown that ErrPs can be detected from single-trial EEG in participants with cerebral palsy, participants with an amputation, and participants with a stroke. The Frontal and Central brain regions were the most important ones, but it was also shown that other brain regions contributed with some discriminative information that increased the classification accuracy slightly. It was also shown that other brain regions beside the Frontal and Central regions could be used to classify ErrPs, this could be important in BCI applications where headsets are used that do not cover the Frontal or Central brain areas e.g., for more aesthetically pleasing headsets. Offline analyses were performed, but the findings should be validated with online error detection in future studies.

## Figures and Tables

**Table 1:** Characteristics of the participants with cerebral palsy. Gender, age, and diagnosis (diplegia or hemiplegia) as well as the affected side, and gross motor function classification system (GMFCS) score are presented.

Participant	Gender	Age (Years)	Diagnose	GMFCS
01	F	12	Diplegia	II
02	F	10	Diplegia	II
03	F	10	Hemiplegia-right	II
04	M	16	Hemiplegia-left	II
05	M	11	Hemiplegia-left	I
06	M	9	Hemiplegia-right	II
07	F	14	Hemiplegia-right	II
08	M	12	Hemiplegia-right	III
09	M	13	Diplegia	III
10	M	15	Diplegia	III

**Table 2:** Characteristics of the participants with an amputation. Gender, age, time since amputation, the level of amputation, and affected side are presented.

Participant	Gender	Age (Years)	Time Since Amputation (Years)	Amputation Level	Amputation Side
01	M	13	3	Hip disarticulation	Left
02	F	45	2	Transfemoral	Left
03	M	32	5	Wrist disarticulation	Right
04	M	27	1	Transfemoral	Right
05	M	30	2	Shoulder disarticulation	Left
06	M	32	5	Transcredial	Right
07	M	53	7	Knee disarticulation	Right
08	M	12	5	Hip disarticluation	Left

**Table 3:** Characteristics of the participants with an amputation. Gender, age, time since amputation, the level of amputation and affected side are presented.

Participant	Gender	Age (Years)	Affected Side	Type of Stroke	Time Since Injury (Days)	Bruunstrom Stage
01	M	48	Right	Haemorrhage	91	II
02	M	55	Right	Ischemic	172	V
03	M	41	Left	Ischemic	70	III
04	M	50	Left	Haemorrhage	90	III
05	M	57	Right	Haemorrhage	52	V
06	M	52	Right	Ischemic	188	V
07	M	24	Left	Haemorrhage	180	IV
08	F	32	Left	Ischemic	25	II
09	F	26	Left	Haemorrhage	20	I
10	M	60	Right	Ischemic	87	IV
11	M	54	Left	Ischemic	220	VII
12	M	46	Left	Ischemic	42	III
13	M	58	Right	Ischemic	84	III
14	M	37	Right	Haemorrhage	36	II
15	M	42	Left	Haemorrhage	118	V
16	M	24	Left	Haemorrhage	45	IV
17	F	26	Right	Ischemic	12	I
18	M	62	Right	Haemorrhage	118	III
19	M	30	Right	Ischemic	60	III
20	F	53	Left	Ischemic	93	IV
21	F	38	Right	Haemorrhage	45	VI
22	F	28	Left	Ischemic	27	V
23	M	45	Left	Ischemic	90	IV
24	M	35	Left	Haemorrhage	17	II
25	M	45	Right	Haemorrhage	280	VI

## Data Availability

The data are not publicly available because of ethics and institutional policy.
